# The Ebola Virus Disease Outbreak in West Africa: A Wake-up Call to Revitalize Implementation of the International Health Regulations

**DOI:** 10.3389/fpubh.2016.00120

**Published:** 2016-06-09

**Authors:** Olushayo Oluseun Olu

**Affiliations:** ^1^World Health Organization, Kigali, Rwanda

**Keywords:** Ebola virus disease, outbreak, West Africa, improved implementation, international health regulations

## Abstract

The 2014/15 Ebola virus disease (EVD) outbreak in West Africa has highlighted the inherent weaknesses associated with the implementation of the International Health Regulations (IHR). In this perspective article, the lessons learnt from the outbreak are used to review the challenges impeding effective implementation of the IHR and to propose policy and strategic options for enhancing its application. While some progress has been achieved in implementing the IHR in several countries, numerous challenges continue to impede its effectiveness, especially in developing countries, such as those affected by the West Africa EVD outbreak. Political and economic sensitivities associated with reporting public health emergencies of international concern (PHEIC), inadequate resources (human and financial), and lack of technical know-how required for implementation of the IHR are weaknesses that continue to constrain the implementation of the regulations. In view of the complex sociopolitical, cultural, and public health dimensions of PHEICs, frameworks, such as the IHR, which have legal backing, seem to be the most effective and sustainable option for assuring timely detection, notification, and response to such events. Renewed efforts to strengthen national and global institutional frameworks for implementation of the IHR are therefore required. Improvements in transparency, commitment, and accountability of parties to the IHR, mainstreaming of the IHR into national public health governance structures, use of multidisciplinary approaches, and mobilization of the required resources for the implementation of the IHR are imperative.

## Introduction: Weak Implementation of the International Health Regulations as a Cause of the West Africa Ebola Outbreak

The International Health Regulations (IHR) is a legal instrument with the purpose of assisting countries to prevent and respond to global public health emergencies, which are a threat to the world’s population (1). The implementation of the regulations, which are binding in 196 countries across the globe, commenced in June 2007. The IHR is a broad legal framework that considers a wide range of public health conditions, provides a rational decision making system for member states on public health emergencies of international concern (PHEIC), and protects the human rights of populations. Its effective implementation is expected to result in the prevention and control of the international spread of diseases, such as the 2014/15 Ebola outbreak in West Africa; this, in turn, should translate into a reduction in the restriction on international travel and trade – a measure instituted by countries in response to disease outbreaks.

The regulations define the rights and responsibilities of signatory countries to build the core capacities required to identify, assess, and report events to the World Health Organization (WHO) and to respond to public health risks, including disease outbreaks. All signatories [known as State Parties (SP)] to the regulations were expected to have built these capacities by the end of 2012, 5 years after the implementation of the IHR, at national, district/intermediate, and community levels. These core capacities are legislation, coordination, surveillance, response, preparedness, risk communication, human resources, and laboratory capacity. The regulations mandate WHO to investigate unofficial rumors of public health events and define procedures for the WHO Director-General to declare an event as a PHEIC.

The 2014/15 Ebola Virus Disease (EVD) outbreak in West Africa is, no doubt, one of the most important PHEICs, severely threatening global health security, recently. While intense disease transmission was largely confined to West Africa, the impact of the outbreak was felt globally. By the time the outbreak was declared over in the last principally affected country, Guinea, on December 29, 2015, 28,637 cases and 11,315 deaths had been reported from 10 countries, making it the biggest outbreak in the history of the disease (2).

Fear of importation of the disease resulted in anxiety and rushed implementation of unnecessary travel restrictions and bans in several countries, causing severe economic loss to both the private and public sectors (3). At the community level, the outbreak wreaked havoc on social and community norms and structures, leading to intense fear and community resistance to outbreak control and preventive interventions (3). In the formal health-care system, the death of hundreds of health-care workers, diversion of human and financial resources from other critical public health programs, and weakened health information management systems totally disrupted health service delivery in the three principally affected countries, Guinea, Liberia, and Sierra Leone (3, 4).

The magnitude, severity, and geographical spread of this EVD outbreak have been attributed to various reasons, including weak health systems, urbanization, rising poverty, deep-rooted health inequity, deterioration in access to social services, ecological changes, and high population mobility, all of which are pervasive in the three principally affected countries (5–7). One major cause often relegated to the background, loudly resonates-weak implementation of the IHR (8).

The outbreak revealed the inherent weaknesses associated with the implementation of the IHR. While the first cases of the outbreak were reported from Guekedou, Guinea, in December 2013, a weak disease surveillance and notification system made it impossible to officially declare the outbreak until March 2014, by which time it had spread to other parts of the country (9). After the declaration of the outbreak by WHO as a PHEIC on August 8, 2015, inadequate capacity for implementation of the IHR and the pervasive weak health systems in the affected countries constrained timely prevention and control of the outbreak (10). Additionally, fear of disease importation resulted in widespread disregard of the IHR.

In this perspective article, the lessons learnt from the Ebola outbreak are used to review the challenges impeding effective implementation of the IHR and to propose policy and strategic options for enhancing its implementation.

## Why is the IHR Not Effective?

While good progress has been made in the implementation of the IHR, several challenges continue to impede the full achievement of its objectives. Key achievements in the implementation of the IHR include designation of national focal points (NFPs) by several SPs, improved communication and synergy between animal and human health sectors through the One Health approach, roll out of effective coordination mechanisms for emergencies, and utilization of early warning systems as tools for early detection and response to emergencies (11).

However, challenges, such as the politically sensitive nature of PHEICs and negative impact of these on international trade, local tourism, economy, and travel, are barriers for countries to promptly report such PHEICs using the IHR. Political sensitivity is often fueled by the media attention generated by the announcement of PHEICs, which may, in turn, result in decreased political popularity of leaders and reduction in their political power (12). This factor may have partially contributed to the late reporting of the EVD outbreak and facilitated its rapid spread.

Poor compliance of SPs to the implementation of the required core capacities for effective application of the IHR is another key challenge (13); by 2012, less than 20% of SPs had fully developed the eight national surveillance and response capacities highlighted in the IHR, which are required for the effective prevention and control of disease outbreaks (8). By 2014, this had increased to a mere third of the countries concerned (14). The low rate of implementation is attributed to a lack of political resolve, inadequate resources (human and financial), and technical know-how required for development of such capacities, especially in low and middle-income countries (LMICs) (11).

Many of the SPs that reported good progress in building these core capacities have not been able to translate them into concrete actions at national and local levels. For instance, while Sierra Leone, one of the principally affected countries, reported more than 70% achievement of five out of the eight core capacities in its 2014 IHR review report, in reality, the actual capacity on the ground during the EVD outbreak was entirely different (15). Conspicuous gaps in the national surveillance system, laboratory capacity, risk communication, and human resources for health hindered an effective response to the outbreak.

World Health Organization has been mandated to monitor compliance with the IHR; however, in reality, the authority of the organization to carry out this function is often undermined by sovereignty of SPs (12). Other challenges to effective implementation of the IHR, which were glaring during the Ebola outbreak include lack of awareness and understanding about the IHR provisions, unavailability of the IHR guidelines and weak coordination of its implementation (11), lack of required capacity and decision making authority of IHR NFPs, lack of a multidisciplinary approach to the implementation of the IHR and limited support, and inadequate technical capacity of LMICs to build their core capacities among other challenges (11).

Poor community engagement and participation in the implementation also challenged the effective implementation of the IHRs. These barriers often fuel community resistance, which is perhaps one of the most important factors responsible for the sustained transmission and scale of the EVD outbreak in West Africa (5).

## Are There Sustainable Alternatives for Assuring Global Health Security?

Several public health surveillance and response systems and networks are available with the aim of complementing the IHR. A number of WHO member states are implementing the Integrated Disease Surveillance and Response system (IDSR) (16), which aim to promptly detect and respond to disease outbreaks. Regional networks, such as the Pacific Island Countries (PIC), have established public health surveillance initiatives, such as the Pacific Public Health Surveillance Network (PPHSN) (17). Evaluation of the IDSR in Uganda demonstrated improved preparedness, surveillance, timely detection, and response to outbreaks, which were associated with implementation of the system (18). However, the results of a similar evaluation conducted in Tanzania were not so promising (19). This evaluation showed weaknesses in capacity for laboratory diagnosis and reporting of epidemic-prone diseases.

In response to the weak implementation of the IHR, the global health security agenda, a joint initiative between a number of United Nations (UN) agencies (including WHO) and 48 countries led by the United States of America (USA), was established in February 2014 to strengthen the capacity of countries to prevent, detect, and respond to pandemics, such as the Ebola epidemic (20). This agenda rallied support to respond to the EVD outbreak and significantly contributed to its eventual prevention and control. Evaluations of the agenda conducted in Georgia, Peru, Portugal, Uganda, and the United Kingdom have also showed good indications of its ability to strengthen global health security (20).

While these initiatives have been instrumental in addressing PHEICs, none of them have the comprehensive legal approach of the IHR. Given the complex sociopolitical, cultural, and public health nature of PHEICs, frameworks with legal backing, such as the IHR, seem to be the most sustainable and effective option for effectively responding to PHEICs (8). Furthermore, the IHR has been tested and found to be valuable in responding to pandemics, such as the 2009 H1N1influenza outbreak (21). The IHR was instrumental in the timely declaration of the outbreak as a PHEIC, issuance of relevant recommendations for its prevention and control, and dissemination of outbreak information among SPs. However, challenges (similar to those experienced in the West Africa EVD outbreak), such as inadequate capacity for outbreak surveillance and response, non-compliance of some SPs with IHR rules on travel restriction and trade ban, and inadequate funding of the IHR were also observed. The IHR has also been shown to be a useful platform for strengthening international cooperation, capacity building, and coordination of emergencies (14).

## Conclusion: Policy and Strategic Options for Enhancing Implementation of the IHR

The impact and magnitude of the West Africa EVD outbreak could have been minimized, if the principally affected countries had been able to effectively implement the IHR core capacities. A strong IHR in these countries would have ensured the capacity for timely identification, notification, and effective response to the outbreak. With growing urbanization, disease vector resistance, climate change, and increased opportunities for interactions between disease vectors and humans, there is no doubt that the next PHEIC is only a matter of when and where, as shown by the ongoing Zika virus, Middle East respiratory syndrome coronavirus, and Yellow fever outbreaks. Drawing on the lessons learnt from the 2014/15 Ebola outbreak, the world should be better prepared for PHEICs in the future.

In the absence of better alternatives and in view of the good results achieved by the IHR in previous PHEICs, a well implemented IHR is the most credible option for improving the response to PHEICs. This highlights the need for renewed efforts to improve the national and global institutional framework for its implementation (8). New and innovative policies, strategies, and concerted efforts are, therefore, required from all SPs to promote and strengthen implementation of the IHR.

Specifically, strong advocacy to the political leadership of SPs should be intensified to galvanize national support for the IHR and to strengthen the institutional arrangements required for its implementation at the national and sub-national levels. Such institutional arrangements should clearly define mechanisms, roles, and responsibility for triggering national action in the event of a PHEIC. Top level policy dialog is also important to realign the provisions of the IHR in the light of the lessons learnt from the EVD outbreak and other recent PHEICs, such as the H1N1 outbreak. The outcome of the ongoing review of the IHR, which was commissioned by the World Health Assembly in May 2015, should provide further insights into why it was not very effective in the prevention and control of the EVD outbreak and propose additional recommendations for improving its efficiency during future outbreaks.

Furthermore, stronger partnerships between health and other national sectors, such as transportation, security, agriculture and education, are required to ensure better collaboration and a coordinated approach to IHR implementation in countries (22). Strengthening of local capacity for implementation of the IHR through better community engagement and participation is also critical. Community participation in outbreak preparedness and response activities would ensure timely detection and reporting of outbreaks, especially in remote communities (such as the case in this EVD outbreak), better understanding of the local context and culture, effective risk communication, and community mobilization for action, which would in turn reduce community resistance.

Improvement in transparency, commitment, objectivity, and accountability of SPs to the IHR through the establishment of mechanisms for facilitating compliance to the provisions of the IHR should be encouraged. SPs should be encouraged to mainstream the IHR into their national public health policies and governance structures; this will empower decision makers and IHR NFPs to fast track its implementation. A multidisciplinary approach to the implementation of the IHR should also be promoted to foster better coordination and collaboration among relevant national sectors.

A combination of negotiation, arbitration, and incentives are proposed as methods of encouraging SPs to promptly report PHEICs to WHO (12, 21, 23). Incentives should include provision of adequate resources to respond to emergencies, respect of the sovereignty of the affected country, and establishment of a global award system to recognize countries that detect, report, and respond to emergencies promptly. Other incentives should include establishment of mechanisms to ensure minimal negative effects of reporting PHEICs and a graded approach to establish a midway for declaration of PHEICs (13).

The causes of failure of the IHR and status of its core capacities should be independently assessed, particularly in countries that are at risk of PHEICs. The findings of such assessments should be used to develop plans to fast track the implementation of the IHR core capacities in these countries. In addition, strengthening the health systems of SPs would complement the IHR capacity to detect and respond to PHEICs (13).

Mobilization of the required resources and technical capacity for implementation of the IHR should be scaled up, especially in LMIC (21); this should be done through the development and implementation of effective national, regional, and global emergency public health work forces and emergency funding policies and strategies. Intensifying awareness about the importance and need for implementation of the IHR is also required in all countries. Sharing of experiences and best practice in the implementation of IHR, among LMICs (who are less able to implement the core capacities), is also recommended. This could be facilitated through south–south and triangular cooperation mechanisms, as was done during the EVD outbreak. For instance, through such arrangements countries, such as Uganda, with good experience in management of EVD outbreaks provided support to manage the outbreak in the principally affected countries, while South Africa and Nigeria (through funding from the European Union) provided laboratory support.

The World Health Assembly should strengthen the mandate and capacity of the WHO Secretariat to independently monitor, assess, and report PHEICs. In this regard, some level of autonomy should be granted to the organization in matters relating to PHEICs (24). Adequate staffing, predictable and timely emergency funding, and a strong operational platform should also be provided to the organization.

Finally, integration and better coordination of similar initiatives, such as the IDSR and PPHSN and the global health security agenda, is required to avoid duplication of efforts and to ensure a more coordinated and collaborative response to PHEICs in the future (16, 17).

## Author Contributions

The author is the sole, corresponding author of the work described in this manuscript. He has designed and implemented the work described.

## Conflict of Interest Statement

The author declares that the research was conducted in the absence of any commercial or financial relationships that could be construed as a potential conflict of interest.
